# Open Prebiotic Environments Drive Emergent Phenomena and Complex Behavior

**DOI:** 10.3390/life9020045

**Published:** 2019-06-03

**Authors:** Nathaniel Wagner, David Hochberg, Enrique Peacock-Lopez, Indrajit Maity, Gonen Ashkenasy

**Affiliations:** 1 Department of Chemistry, Ben-Gurion University of the Negev, Beer Sheva 84105, Israel; indrajit.maity@makro.uni-freiburg.de (I.M.); gonenash@bgu.ac.il (G.A.); 2 Department of Molecular Evolution, Centro de Astrobiología (CSIC-INTA), Ctra Ajalvir Km. 4, 28850 Torrejón de Ardoz, Madrid, Spain; hochbergd@cab.inta-csic.es; 3 Department of Chemistry, Williams College, Williamstown, MA 02167, USA; epeacock@williams.edu; 4 Present address: Institute for Macromolecular Chemistry, Albert Ludwigs University of Freiburg, D-79104 Freiburg, Germany

**Keywords:** systems chemistry, catalytic networks, prebiotic replicators, complexification, emergence

## Abstract

We have been studying simple prebiotic catalytic replicating networks as prototypes for modeling replication, complexification and Systems Chemistry. While living systems are always open and function far from equilibrium, these prebiotic networks may be open or closed, dynamic or static, divergent or convergent to a steady state. In this paper we review the properties of these simple replicating networks, and show, via four working models, how even though closed systems exhibit a wide range of emergent phenomena, many of the more interesting phenomena leading to complexification and emergence indeed require open systems.

## 1. Introduction

Complex systems in nature are most appropriately modeled as open networks [1], where energy, raw materials and reactants are continuously being pumped in, and reactants and products are prone to dissipation and decay. The ecosystem, for example, exists far from equilibrium, in a continuous state of flux [2,3]. Living organisms, in order to stay alive, are constantly importing nutrients and energy in various forms and exporting waste products and heat. Individual cells within organisms also interact with their local environment. Similarly, many natural and artificial non-living systems, such as rivers, fountains and reactors, exist far from equilibrium, at times exhibiting steady-state behavior but always interacting with their environments [4].

Studying synthetic networks exhibiting emergent phenomena is at the focus of Systems Chemistry research [5,6,7,8]. The design and analysis of such networks may shine light on early chemical evolution processes that led to emergent properties in prebiotic environments, while at the same time they might be useful for the construction of synthetic cells. Simple (prebiotic) replicators and networks may be open or closed, namely, interacting with their environments, or alternately, chemically and thermally isolated. Both systems have therefore been studied, enabling precise modeling and rigorous analysis, and allowing crucial insight into the minimal requirements for various emergent phenomena. 

In this paper, we review several of the simple replicators and catalytic networks we have previously investigated as prototypes for modeling replication, complexification and emergence. We discuss each model, its properties and emerging phenomena. In order to analyze the unique features of each model, we begin with the simplest model and proceed towards increasing complexity. Furthermore, we will attempt to answer a broader query: when using these simple prebiotic models, is it necessary to use open systems, or are closed systems sufficient in order to observe many of the interesting phenomena that emerge from these models?

## 2. Minimal Self-Replication and Catalytic Reaction Networks

Catalytic reaction networks, based on simple models of minimal self-replication and higher order catalysis, consist of molecular arrays interconnected by autocatalytic and cross catalytic pathways among reactants and templates [9,10]. These synthetic networks have been studied in order to unravel complex system behavior, and can serve as “bottom-up” models for the design and understanding of molecular evolution and emergent phenomena [11,12]. In the context of Systems Chemistry [13,14,15,16,17], catalytic reaction networks have been studied theoretically and computationally [18,19,20,21,22,23,24,25,26,27,28,29,30,31,32,33], and have been realized experimentally in several distinct chemical systems, including nucleic acids [34,35,36,37,38,39,40], fatty acids [40], peptides [38,41,42,43,44,45], organic abiotic molecules [40,46,47,48] and enzymatic networks [49,50]. Subsequent studies have shown how small catalytic networks may be designed to perform Boolean logic operations [51,52,53,54,55,56,57,58,59,60,61,62,63,64,65,66,67], and to mimic computational modules [31] and network motifs [68,69], and they may further display oscillations and circadian rhythms [70,71], bistability and bifurcations [72,73,74] and control competition and cooperation [75,76].

The models we discuss throughout the paper were developed to account for our experimental work, in which the basic replication motifs are driven by coiled coil peptides assembly, self-replication and/or cross catalysis [53,72,75,76]. Nevertheless, the same theoretical and simulation framework can be used to probe other minimal replication systems, such as those developed with short nucleic acid sequences or abiotic molecules. Our peptide-based replication system has been described in detail in our previous papers and reviews [10,11,12,31,32,67,70,71,77]; briefly, during replication, a dimeric peptide assembly serves as the template for association of two shorter precursors, which then, due to their close proximity and end-groups positioning, react and ligate. In the case of an autocatalytic process, the product of this ligation is the same as (at least one of) the template monomeric peptides, while in a cross catalytic scenario the product is a peptide of the same length as the template monomers but differs in its sequence (a ‘mutant’). Note that in several places along the paper we mention modifications made to the basic replication systems in order to facilitate a specific function; these changes to the models are also explained in more detail in the original papers.

The most basic model consists of minimal self-replication, consisting of two fragments (we use the letters *E* and *N* since many experimental systems, such as peptides, are composed of electrophilic and nucleophilic fragments) termed *E* and *N* joining together to form a template product *T*, either directly (Equation (1)), or through template-assisted ligation via first order catalysis (Equation (2)) or second order catalysis (Equation (3)):

(1) $$E+N\overset{g}{\to }T$$

(2) $$E+N+T\begin{array}{c} \overset{a}{\to }\\ \underset{\bar{a}}{\leftarrow } \end{array}ENT\overset{b}{\to }TT\begin{array}{c} \overset{d}{\to }\\ \underset{\bar{d}}{\leftarrow } \end{array}T+T$$

(3) $$E+N+TT\begin{array}{c} \overset{a}{\to }\\ \underset{\bar{a}}{\leftarrow } \end{array}ENTT\overset{b}{\to }TTT\begin{array}{c} \overset{f}{\to }\\ \underset{\bar{f}}{\leftarrow } \end{array}TT+T\begin{array}{c} \overset{d}{\to }\\ \underset{\bar{d}}{\leftarrow } \end{array}T+T+T$$

The rate constants, describing ligation, diffusion and dissociation, are contained in the equations. Since *T* itself serves as a catalyst in its own formation, this is a form of self-replication.

Since we have previously shown how the behavior of second order catalysis is richer, more versatile [32] and more relevant to actual experiments [73], and furthermore, how higher order catalytic reactions are necessary for processes of emergence, evolution, self-organization and complexification [77]—as will be elaborated on below—we will concentrate here mainly on the second order replication and network pathways.

Equations (1) and (3) can be expanded to describe a replication network:

(4) $$E_{i}+N\overset{g_{i}}{\to }T_{i}$$

(5) $$E_{i}+N+T_{j}T_{k}\begin{array}{c} \overset{a}{\to }\\ \underset{{\bar{a}}_{ijk}}{\leftarrow } \end{array}E_{i}NT_{j}T_{k}\overset{b_{i}}{\to }T_{i}T_{j}T_{k}\begin{array}{c} \overset{f_{ijk}}{\to }\\ \underset{\bar{f}}{\leftarrow } \end{array}T_{i}+T_{j}T_{k}\begin{array}{c} \overset{d_{jk}}{\to }\\ \underset{\bar{d}}{\leftarrow } \end{array}T_{i}+T_{j}+T_{k}$$

In the above simple network, several *E_i_*’s react with a common *N* (without loss of generality), producing several products *T_i_*. The *i* = *j* = *k* case is autocatalytic, since *E_i_*’s are being catalyzed by *T_i_*’s to produce more *T_i_*’s. On the other hand, *i* ≠ *j,k* describes cross catalytic pathways, since the *T_i_*’s being formed from the *E_i_*’s are being catalyzed by *T_j_*’s and/or *T_k_*’s. As before, each product can also be produced by a slower background reaction (Equation (4)).

As stated above, very similar models can be used to study minimal replication processes based on nucleic acid or organic abiotic molecules. While the high order aggregation states existing for protein assembly facilitated the second order replication discussed here, only first order replication was demonstrated so far using short DNA sequences or RNA ribozymes. Still, one can imagine a scenario in which DNA duplexes serve as templates and/or catalysts for the formation of a third molecule, presumably even an RNA ribozyme.

As an additional twist to the original model, when appropriate, the ligation steps in the above reactions can be made reversible:

(6) $$E_{i}+N\begin{array}{c} \overset{g_{i}}{\to }\\ \underset{{\bar{g}}_{i}}{\leftarrow } \end{array}T_{i}+S$$

(7) $$E_{i}+N+T_{j}T_{k}\begin{array}{c} \overset{a}{\to }\\ \underset{{\bar{a}}_{ijk}}{\leftarrow } \end{array}E_{i}NT_{j}T_{k}\begin{array}{c} \overset{b_{i}}{\to }\\ \underset{{\bar{b}}_{i}}{\leftarrow } \end{array}T_{i}T_{j}T_{k}+S\begin{array}{c} \overset{f_{ijk}}{\to }\\ \underset{\bar{f}}{\leftarrow } \end{array}T_{i}+T_{j}T_{k}+S\begin{array}{c} \overset{d_{jk}}{\to }\\ \underset{\bar{d}}{\leftarrow } \end{array}T_{i}+T_{j}+T_{k}+S$$

Here, the ligation reactions also produce *S*. Note that in many cases we monitor the values of *t_i_*, which are the net sums of the *T_i_* concentrations in all aggregation states.

This reversible scheme has also been implemented experimentally in peptide networks by replacing an amide bond with a thioester bond [75] and using free thiol molecules *S* to drive the reversible ligation. Since the template trimer *TTT* is much more stable than the template monomer *T* [72], the reverse ligation rate constant $\bar{b}$ is practically zero in the experimental system.

**Scheme 1:**
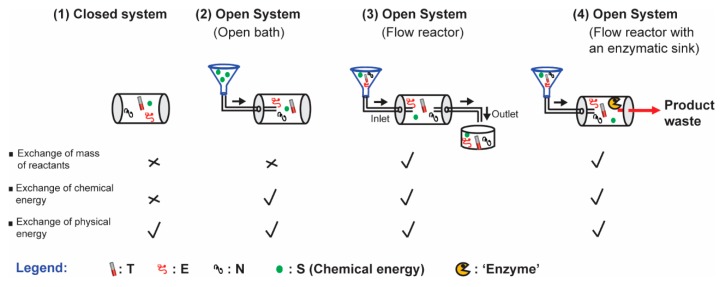
Four working models for open or closed networks that correspond to four different experimental conditions. The reactants *E* and *N* and product *T* directly participate in the reactions, while the chemical species represented by *S* (“chemical energy”) and the enzyme do not directly take part; *S* plays an important role in keeping the reactants from unwanted decay, and the enzyme enables an enzymatic decay of the product only.

We now describe several options for modeling each network as open or closed, as shown in Scheme 1, which also correspond to four different experimental (and possibly prebiotic) conditions:

(1) A closed system, consisting of fixed amounts of starting reactants that ligate and converge to equilibrium.

(2) An “open bath” configuration, consisting of reactants that ligate (reversibly) in a stoichiometrically closed system that is continuously fueled by energy (and a large excess of certain molecules).

(3) An open flow reactor, consisting of a continuous flow of reactants in a chemostat configuration or a CSTR (continuously stirred tank reactor), where the intake flow (of starting materials) is at the same rate as the clearance (of products and unreacted starting materials).

(4) An open flow reactor in a different mode, consisting of a continuous intake flow of reactants and enzymatic degradation of the products only.

These four models begin with the most simple, closed system and progress towards increasing openness and complexity.

Remarkably, we note that chemical thermodynamics imposes constraints upon the closed system [78,79]. If we take the reactions of Equations (6) and (7) at equilibrium, we obtain

(8) $$\frac{\bar{g}\;a\;b\;f}{g\;\bar{a}\;\bar{b}\;\bar{f}}=1$$

Since our system is described by mass-action kinetics, these constraints on the reaction rate constants are independent of the concentrations, and therefore should hold true not only at equilibrium, but also far away from equilibrium.

**Scheme 2:**
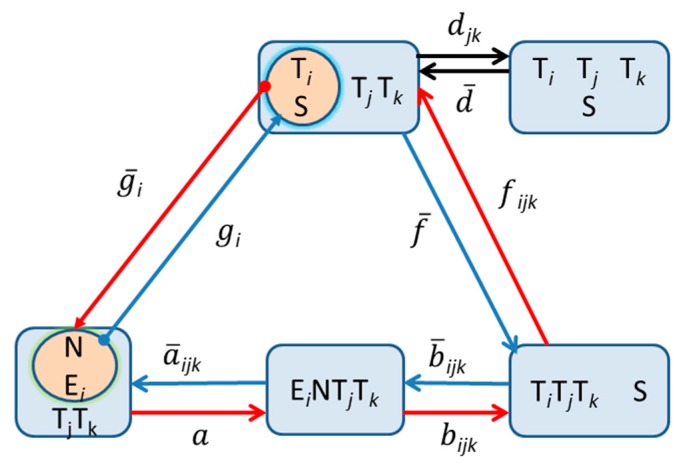
The fully reversible replication network of Equations (6) and (7), emphasizing the closed cycles involved in the corresponding Wegscheider condition (Equation (9)), for which the product of the equilibrium constants around any closed reaction pathway must be equal to unity. The clockwise sense is depicted by the blue pathway, whereas the counter-clockwise path is indicated in red. Note that since the dissociation of template dimers into single templates is not involved in these cycles (see upper right corner), the corresponding rate constants $\bar{d}$ and *d*_jk_ are not constrained.

A similar constraint exists for the rate constants in the network case. For each reactant *i*, it can be shown, using the Wegscheider Condition [80], that the product of the equilibrium constants around any cycle (see Scheme 2) must be equal to unity:

(9) $$\frac{{\bar{g}}_{i\;}a\;b_{i}\;f_{ijk}}{g_{i}\;{\bar{a}}_{ijk}\;{\bar{b}}_{i}\;\bar{f}}=1$$

This means that $\bar{b}$ cannot be zero unless $\bar{g}$ is also zero. Therefore, the closed system must either be fully irreversible, or have both non-zero $\bar{b}$ and $\bar{g}$. The suggested semi-reversible model using $\bar{g}$ > 0 and $\bar{b}$ = 0 cannot exist in a closed system. In fact, the corresponding experimental systems are not closed, but are constantly being pumped in with TCEP (tris(2-carboxyethyl)phosphine) as a reducing agent [72,76], making them into “open bath” configurations.

## 3. Emergent Phenomena

We now describe several representative examples of emergent phenomena that appear in these systems and networks.

### 3.1. Cooperation and Competition

As was shown earlier for nucleic acid replication [18] and later on for peptide-based replication [10], the rate of product formation is a critical issue in modeling replication and evolutionary dynamics. Even the simplest first order minimal self-replication in a closed system can display varying rates of growth, depending on its parameters and rate constants, ranging from exponential growth to parabolic growth. In a network of replicators competing for resources, exponential growth leads to “survival of the fittest,” while parabolic growth leads to “coexistence” [10,18,19,20,21,77]. 

**Figure 1:**
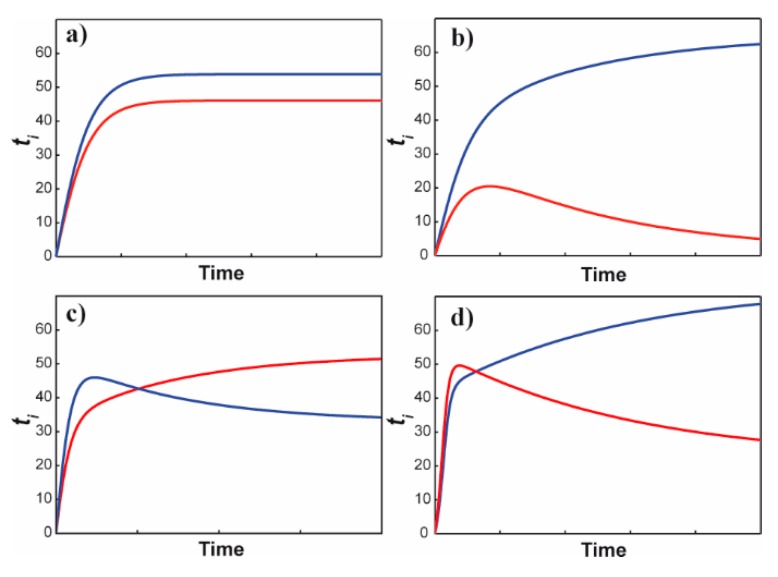
Computational results for two competing replicators, *T*_1_ (blue) and *T*_2_ (red), in an “open bath” binary network. The distinct kinetic behaviors show various expressions of product selectivity. The results were computed using our mass-action kinetic simulation [31] according to Equations (6) and (7) with $\bar{b}$ = 0 for all templates. In all cases, the blue is a more efficient replicator than the red. (**a**) Irreversible case, i.e., all $\bar{g}$ = 0; (**b**) With the blue replicator more stable than the red, i.e., $\bar{g}$_1_ < $\bar{g}$_2_, displaying monotonic growth in the blue template with a reversal in the growth of the red template; (**c**) With the blue replicator less stable than the red, i.e., $\bar{g}$_1_ > $\bar{g}$_2_, displaying a crossover effect in the growth of the templates and a reversal in the blue template; (**d**) With the blue replicator more stable than the red, but with a third external template catalyzing the formation of the red, displaying an opposite crossover effect and a reversal in the red template. Reproduced from [76] with permission from Wiley.

**Figure 2:**
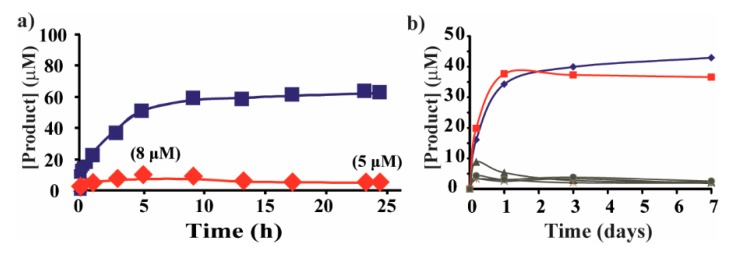
Experimental results for two competing reversible peptide replicators. (**a**) The blue replicator is more efficient and more stable than the red replicator, displaying monotonic growth in the blue template with a reversal in the growth of the red template. Reproduced from Ref. 75 with permission from the Royal Society of Chemistry. (**b**) The blue replicator is less efficient but more stable than the red, displaying a crossover effect in the growths of the templates and a reversal in the red template; this experiment was carried out in the presence of several other inefficient replicators (gray lines). Reproduced from [76] with permission from Wiley.

We can model a simple binary replication network using the reactions of Equations (6) and (7) for two distinct autocatalytic templates. Since the templates compete for common resources (i.e., the common *N*), their behaviors will be interdependent even without any mutual cross catalysis. The behaviors of the relative templates can be differentiated by different initial concentrations, different efficiency (i.e., distinct values of $\bar{a}$, *f* and *d* or *b* and *g*), different stability (i.e., distinct values of $\bar{g}$ and $\bar{b}$) or by using separate external cross catalytic external templates. The distinct kinetic behaviors may result in various expressions of product selectivity. In Figure 1, we show computational results for two competing distinct replicators in an “open bath” network (Model #2 in the above description), i.e., with $\bar{b}$ = 0. These results exhibit interesting effects of crossover and reversal and product selectivity, which are examples of more complex cooperative and competitive network behavior. Actual experiments with peptides have demonstrated these types of behavior, as seen in the two examples displayed in Figure 2 [75,76].

**Figure 3:**
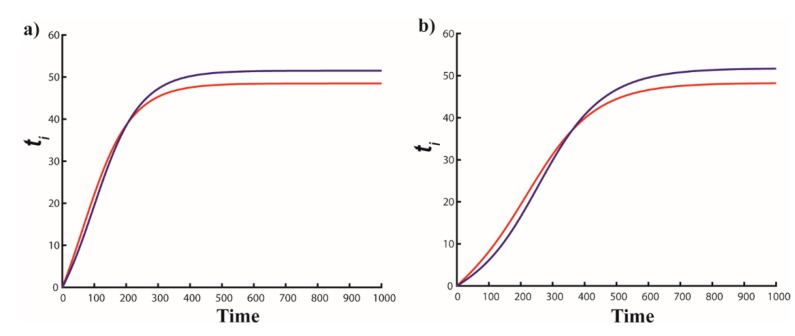
Computational results for two competing reversal replicators in a closed binary network. The results were computed using our mass-action kinetic simulation [31] according to Equations (6), (7) and (9). In both cases the blue replicator is more efficient and more stable than the red replicator. (**a**) With the red replicator beginning with a higher initial concentration; (**b**) With a third external template catalyzing the formation of the red replicator. The results show a minimal crossover effect without the reversal effect and practically no product selectivity.

Interestingly, for similar scenarios in a closed reversible system, where the reverse ligation rate constants follow Equation (9), we obtained the crossover effect without the reversal effect and practically no product selectivity, as can be seen in Figure 3.

### 3.2. Chemical Computation and Logic Operations

**Figure 4:**
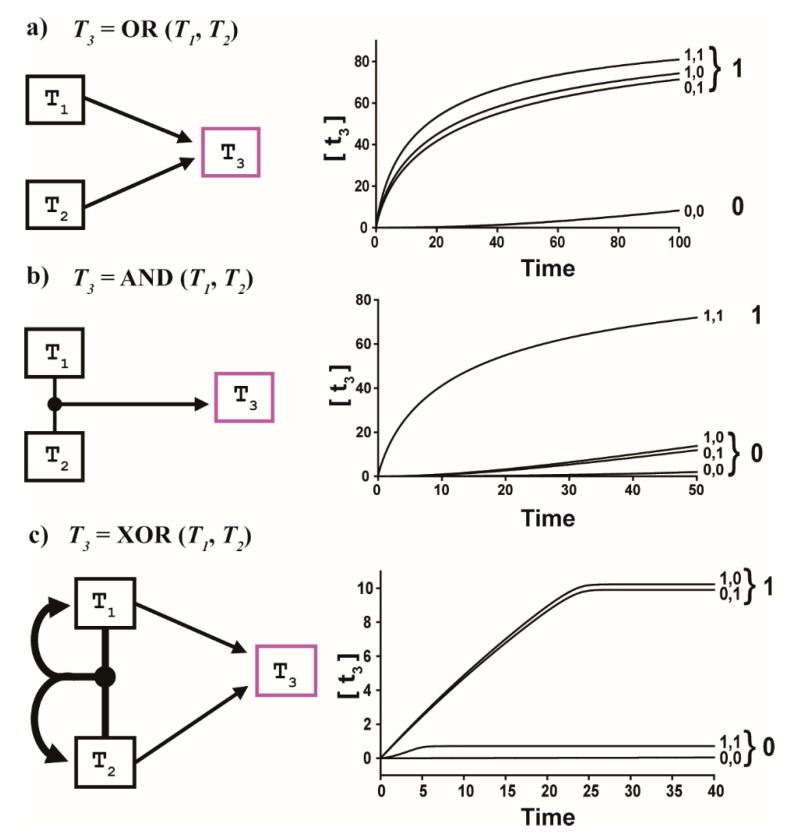
Three two-input logic gates produced computationally using a closed ternary catalytic network. Each graph shows the results of *T*_3_ production for four possible configurations of initial *T*_1_ and *T*_2_. Reproduced from [31] with permission from Wiley.

**Figure 5:**
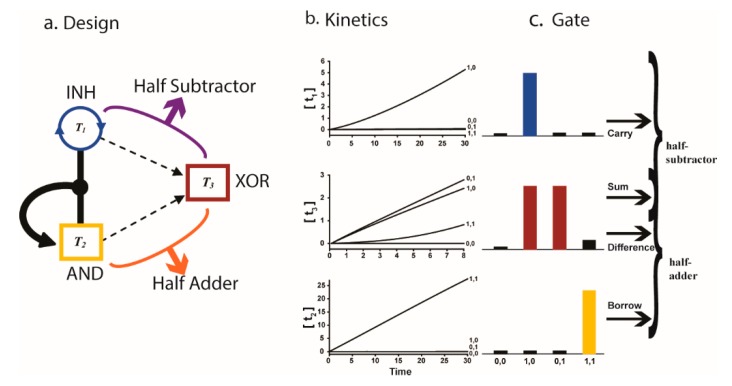
Arithmetic modules—half substractor and half adder—produced computationally using a closed ternary catalytic network. The graphs show the results of *T*_1_, *T*_2_ and *T*_3_ production for four possible configurations of initial *T*_1_ and *T*_2_. Reproduced from [31] with permission from Wiley.

Simple closed ternary networks have succeeded in producing logic gates and arithmetic modules, both computationally [11,12,31] and experimentally [53]. These computational abilities are examples of complex and cooperative network behavior that are considered to be related to complexification, emergence and the collective behavior of living systems. Several representative examples and computational results, based on the reactions of Equations (4) and (5), are displayed in Figure 4 and Figure 5. In the OR gate, both *T*_1_ and *T*_2_ are capable of cross catalyzing *T*_3_, so the initial presence of either of these two templates will result in the production of *T*_3_. In the AND gate, only the heterodimeric template *T*_1_*T*_2_ is capable of catalyzing *T*_3_, so only the initial presence of both of these two templates will result in the production of *T*_3_. The Exclusive OR (XOR) gate is set up like the OR gate, with the additional strong catalytic pathways that allow the heterodimeric template *T*_1_*T*_2_ to catalyze *T*_1_ and/or *T*_2_; in this case, the initial presence of both of these templates will actually prevent the production of *T*_3_. In the arithmetic module (Figure 5), a special network setup allows simultaneous AND, XOR and INHIBIT (i.e., *T*_1_ and not *T*_2_) gates, resulting in a half-adder and a half-subtractor—computational circuits that implement binary arithmetic. 

An experimental AND gate has also been constructed employing light as an inducer, and using a caged random coil template that is catalytically inactive; shining light uncages the peptide, facilitating its folding into a coiled coil helical structure, and driving the replication and computational functionality [81]. Note that this system is essentially open, requiring the input of an external energy source.

**Figure 6:**
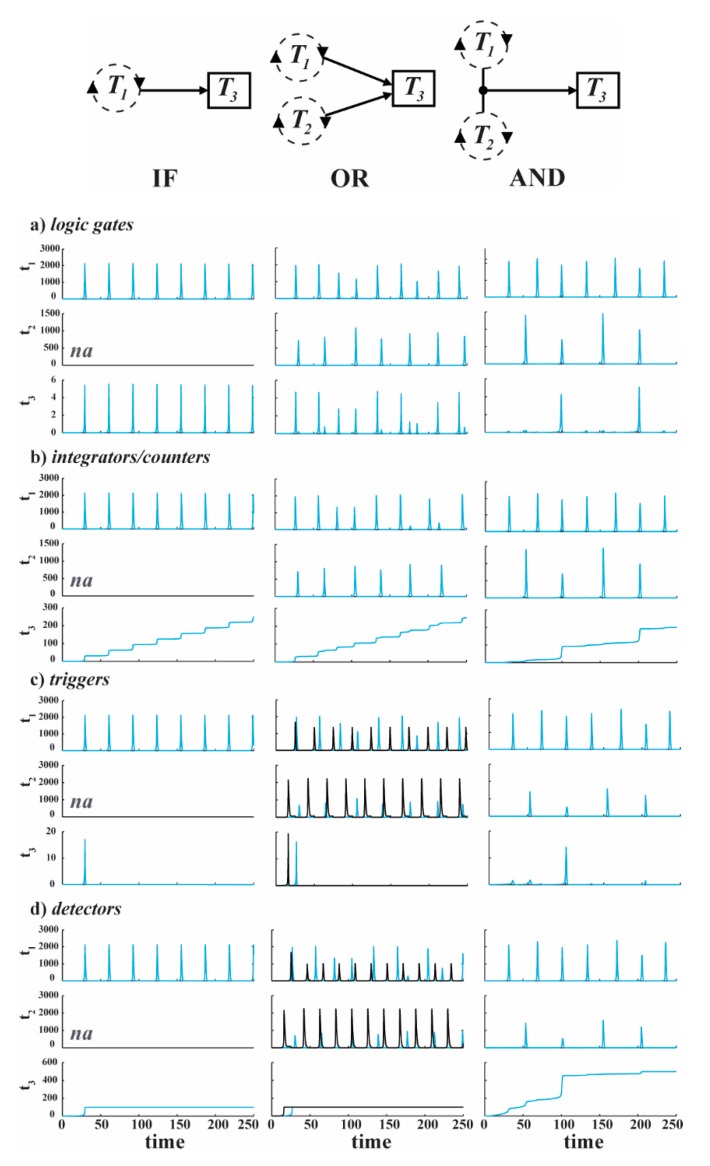
Coupled oscillations in ternary replication networks. The columns corresponding to the IF, OR and AND Boolean logic functions. Oscillations in the intakes of *T*_1_ and/or *T*_2_ are coupled to the production of *T*_3_, resulting in oscillating logic gates (**a**), Integrators/Counters (**b**), Triggers (**c**), and Detectors (**d**). Reprinted with permission from [70], Copyright (2015) American Chemical Society.

By opening up the system with an open flow reactor and enzymatic degradation of the products (Model #4), we have computationally produced “oscillating logic gates,” producing integrators/counters—that integrate or count the appropriate input signals; triggers—one-time non-repeating output signals upon arrival of the first appropriate input signal; and detectors—one-time indicators that an appropriate input signal has arrived, as displayed in Figure 6. The network connectivities of the OR and AND gates are similar to the corresponding gates of Figure 4 above (the IF gate is just like the OR but with only one input), but the inputs *T*_1_ and *T*_2_ are now oscillating intakes, resulting in a more dynamic logic being produced in *T*_3_.

**Figure 7:**
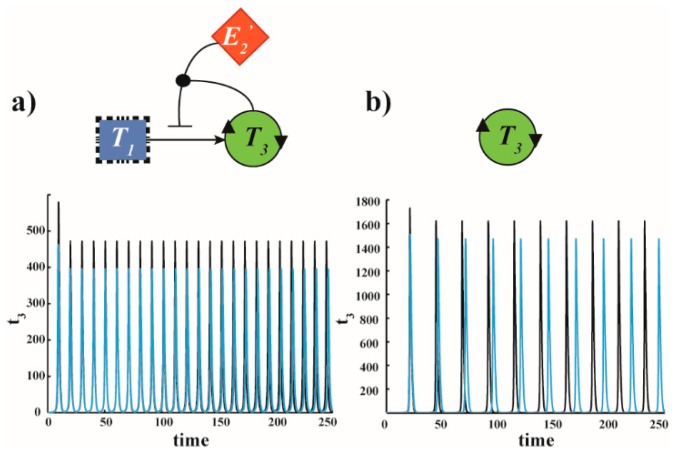
(**a**) Proposed Circadian Network, based on the circadian clock of *S. elongatus*, consisting of autocatalytic, cross catalytic and inhibitory negative feedback pathways. The output frequencies of oscillation are independent of the intake and decay rates. (**b**) A simple autocatalytic network, without any internal stabilizing mechanism, whose frequencies of oscillation vary significantly with differing intake and decay rates. Each case compares two sets of intake and decay rates that differ by 20%. Reprinted with permission from [70], Copyright (2015) American Chemical Society.

Additionally, opening up these small networks has enabled us to mimic the mechanisms of biological oscillators. For example, the circadian clock of *S. elongatus*, based on a core of only three proteins *KaiA*, *KaiB* and *KaiC* [82,83,84], can be modeled by the simple ternary network of Figure 7, consisting of autocatalytic, cross catalytic and inhibitory negative feedback pathways. This simple configuration contains a stabilizing mechanism that effectively functions as an internal clock, leading to constant frequencies of oscillation that are independent of the intake and decay rates. The robustness of this network enables it to function as a biological oscillator in living systems that require internal clocks. In Figure 7, we compare the oscillations of this proposed Circadian Network with those of a simple autocatalytic network, without any internal stabilizing mechanism, whose frequencies of oscillation vary significantly with differing intake and decay rates [70,71].

### 3.3. Bistability and Bifurcations

**Figure 8:**
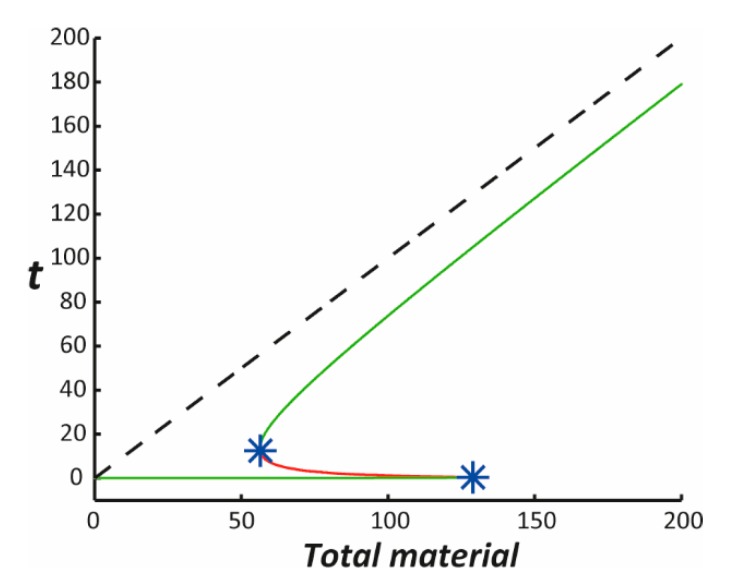
Numerically computed bifurcation diagram, showing steady state concentrations as a function of total material. The two green curves show stable solutions, while the red curve is an unstable solution. The blue stars are the bifurcation points; within their range there exist two stable steady state solutions, one at a very low concentration and the other relatively high. For total material below or above the bifurcation points, there exists only one steady state solution. The red curve is also the demarcation line for the initial concentrations, determining which stable solution will eventually be reached. Reproduced from [73] with permission from Wiley.

**Figure 9:**
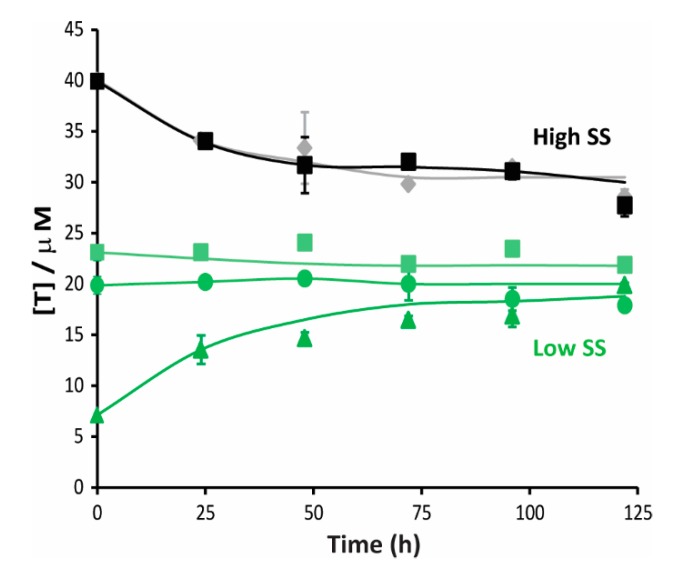
Experimental template concentrations as a function of time. showing a clear separation between the lower and the higher steady states, depending solely on the initial concentration. In this example, initial concentrations of 40 μM or above lead to the higher steady state, while initial concentrations of 23 μM or lower lead to the lower steady state. Reproduced from [72] with permission from Wiley.

**Figure 10:**
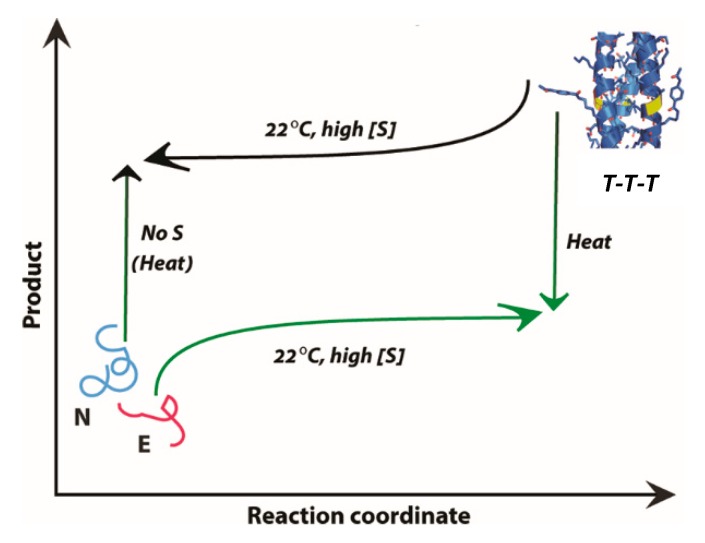
Schematic description of the experimental bistable system. The high steady state is characterized by a high concentration of template in trimer form (*T-T-T*), while the low steady state is dominated by high concentrations of the reactants *E* and *N*. The system can be switched between the two steady states by applying heat or by initiating the forward reaction in the absence of *S* (thiol) at higher temperature. Reproduced from [72] with permission from Wiley.

Numerical steady-state solutions for reversible one-template second order catalysis, with $\bar{g}$ > 0 but $\bar{b}$ = 0, have displayed bistability and bifurcations, i.e., regions allowing for two possible stable steady states. The system will converge to one of these states, depending on the initial concentrations (Figure 8). Subsequently, bistability was also found to occur experimentally using the peptide replication networks (Figure 9), and an important experiment was devised that enabled switching from one steady state to the other (Figure 10) [72]. In our “open bath” simulations, bistability was never found in the first order systems, and it was proven mathematically that bistability requires at least second order catalysis for these systems [73]. Significantly however, bistability has also been found in open flow (Model #3) first order systems (for example [74]; see also additional references in [73]).

**Figure 11:**
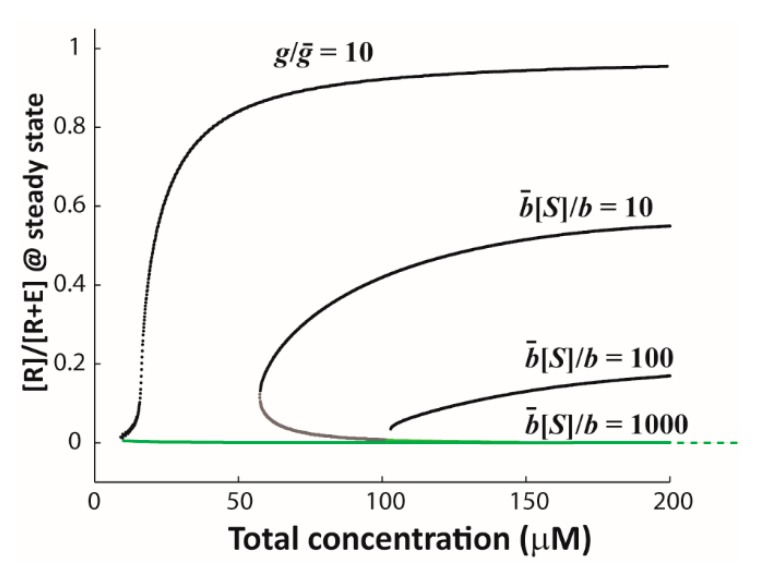
Steady state (normalized) concentrations as a function of total material, computed under different numerical parameters. Increasing $\bar{b}$ leads to the loss of the bistability. Reproduced from [72] with permission from Wiley.

Further analysis of the conditions leading to bistability have shown that if $\bar{b}$ > 0, the bistability decreases (Figure 11) [72]. If $\bar{b}$ is set according to Equation (9), there is no bistability. This is well understood in light of our discussion above, since the system is then closed and must eventually lead to one equilibrium solution. Bistability requires open systems, as summarized by Epstein and Pojman: “Because the alternative states of a multi-stable system are all stable over time, thermodynamics imposes that multi-stability is fundamentally an energy-consuming, out-of-equilibrium process” [85]. Therefore, the choice of $\bar{g}$ > 0 with $\bar{b}$ = 0 crudely approximates an open system for which it might be possible to establish a unidirectional flux of matter in a cyclic reaction path, and so overcome the condition imposed by Wegscheider. Essentially, we are forcing the system to be open, but since we are not using an intake of materials, this corresponds to the “open bath” stoichiometrically closed system. The corresponding experimental systems are also not closed; they are kept with high thiol concentration and are constantly being pumped in with TCEP as a reducing agent [72,75], which prevents the reactants and products from decaying due to hydrolysis and other side reactions that are destructive to the autocatalytic process [73].

### 3.4. Chemical Oscillations

The original Belousov–Zhabotinsky Reaction [85] was produced in a closed system when operating in the early stages of material consumption far from equilibrium. We have not succeeded in producing oscillations in closed catalytic networks—neither experimentally nor computationally. While sustained oscillations clearly require open systems, damped oscillations in closed systems are not thermodynamically prohibited [86]. Even so, we have not observed them, not even in the stoichiometrically closed “open bath” configuration; at most, we observe one or two reversals in the template concentration (e.g., see above in the section on “Cooperation and Competition” and in Figure 1). Since living organisms are replete with rhythmic and oscillatory behavior at all levels, to the extent that oscillations have been termed a “defining attribute of life” [87], this further underscores the important connection between life and open systems.

**Figure 12:**
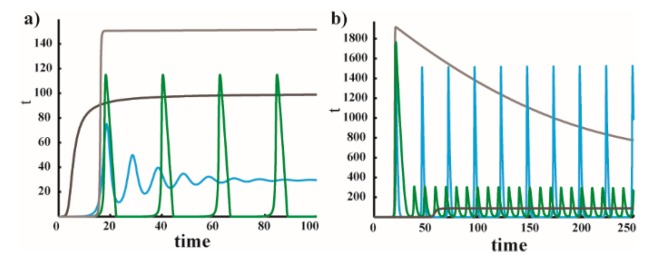
Product formation as a function of time for (**a**) first order autocatalysis and (**b**) second order autocatalysis. The graphs display product formation under the following conditions: a closed system without any intake or sink (dark gray); open systems with intakes for *E* and *N*, and linear (blue), enzymatic (green), and constant (light gray) sinks for *T*, respectively. Reprinted with permission from [70], Copyright (2015) American Chemical Society.

Oscillations can be produced in an open flow reactor with a continuous intake flow of reactants and degradation of products (Models #3 and #4). If the product decays linearly, first order systems can produce only damped oscillations, while second order systems can also produce sustained oscillations. On the other hand, if the product decays enzymatically, both first order and second order systems are capable of exhibiting sustained oscillations. This has been proven theoretically [26,27,28,29] and demonstrated computationally (see Figure 12) [70].

## 4. Conclusions

In this paper we have looked at simple replicating networks, and have shown how multiple examples of emergent phenomena are critically dependent on the degree to which the model systems are either open or closed. We have shown that closed systems can exhibit a range of emergent phenomena. On the other hand, many of the more interesting phenomena leading to complexification and emergence, such as bistability, bifurcations and oscillations, indeed require open systems! Apparently, the degree of openness also plays a role, as seen by comparing the fully open systems (Models #3,4) with the partially open systems (Model #2).

One of the key differences between the closed and open systems is the reversibility of all the reactions and its impact on the outcome. In the closed systems, all the reactions are reversible (at least in principle) and the only final state is equilibrium. Recalling that a closed system is generally not isolated thermally and heat exchange is allowed, we can control the temperature and change the value of the equilibrium constant, but if all the reactions are kept reversible, we again only have equilibrium as the final state.

Most models that show complex dynamics have at least one or two irreversible steps. In the case of the Belousov–Zhabotinsky Reaction, although closed, we observe transient oscillations in the concentrations of the catalysts in a system where the forward rate constants are much greater than the backward rate constants. Therefore, in a model, we can just neglect those backward processes by making several mechanistic steps irreversible (provided there is no Wegscheider condition that might get violated). In our simpler models, however, the reverse ligation steps cannot be neglected if we want to keep the system closed. Accordingly, some of our conclusions regarding the limitations of closed systems may be true only for simple, prebiotic models, and may not extend to more complex systems. This is also consistent with the fact that higher order catalysis or enzymatic decays (i.e., increased complexity) are more adept at producing oscillating behavior.

Open systems are characterized by heat and/or mass exchange, although most models ignore heat exchange when the range of experimental temperature is small and close to room temperature. A chemostat or CSTR can be used as a model, but any exchange of energy with the system that yields a chemical change will do. For example, in the case of dimerization with a negligible reverse rate constant, we can irradiate the sample and break the dimer, so one effectively changes the reverse rate constant.

In the case of bistability, we maintain a high concentration of active (thiol) molecules by regularly adding a reducing reagent. Thus, we have an open system. Using the minimal model, we argue that the excess thiol makes some reactions reversible, except for the catalytic ligation, so we model the open system by keeping one reaction irreversible. Furthermore, we have proven that first order self-replication cannot sustain bistability, but second order can. The numerical solutions show clearly the high product and the high reactant states of the system. We can even argue that the second order self-replication divides the network into two subnetworks of chemical reactions controlled by the irreversible reaction, and the system gets trapped in one of the subnetworks. If we make the system fully reversible, we lose the bistability.

This can also be understood as follows: bistability requires a mismatch between forward and reverse processes. In our case, this was achieved by a system with template-assisted ligation, but with reverse ligation possible only in the slower background reaction. In a closed system, the template-assisted ligation also becomes reversible, and the mismatch is removed.

In an open semi-batch system with an enzymatic sink, we find complex dynamics for the first and second order self-replication. The sink may balance the input and shift the non-equilibrium steady state and therefore modify its stability, thus producing oscillations. We can interpret the enzymatic sink as a reaction that degrades the product into compounds that can be removed from the reactor, avoiding any accumulation. Although the enzymatic sink may not be an experimental option, in prebiotic conditions a catalytic surface can play the same role, because a surface, as well as an enzyme, can be saturated by the reagents.

In summary, we have demonstrated the crucial role of open systems in driving complexity and emergence, by looking at simple replicating networks using four working models for open or closed networks. We have also shown how studying synthetic replication networks has enabled us to mimic many aspects of life-like behavior. Considering the central role played by catalytic order in driving emergent phenomena [77], there may be a more general rule here: emergence can be driven by either increasing openness or by increasing complexity.
